# Immune mechanisms in vulvodynia: key roles for mast cells and fibroblasts

**DOI:** 10.3389/fcimb.2023.1215380

**Published:** 2023-06-08

**Authors:** Elena Tonc, Gloriah K. Omwanda, Kevin Arnold Tovar, Xiu Mei Emma Golden, Devavani Chatterjea

**Affiliations:** Biology Department, Macalester College, St. Paul, MN, United States

**Keywords:** vulvodynia, chronic pain, mast cells, innate inflammation, fibroblasts, allergies

## Abstract

Vulvodynia is a debilitating condition characterized by painful sensitivity to touch and pressure in the vestibular tissue surrounding the vaginal opening. It is often a “diagnosis of exclusion” of idiopathic pain made in the absence of visible inflammation or injury. However, the association between increased vulvodynia risk and a history of yeast infections and skin allergies has led researchers to explore whether immune mechanisms of dysregulated inflammation might underlie the pathophysiology of this chronic pain condition. Here we synthesize epidemiological investigations, clinical biopsies and primary cell culture studies, and mechanistic insights from several pre-clinical models of vulvar pain. Taken together, these findings suggest that altered inflammatory responses of tissue fibroblasts, and other immune changes in the genital tissues, potentially driven by the accumulation of mast cells may be key to the development of chronic vulvar pain. The association of increased numbers and function of mast cells with a wide variety of chronic pain conditions lends credence to their involvement in vulvodynia pathology and underscores their potential as an immune biomarker for chronic pain. Alongside mast cells, neutrophils, macrophages, and numerous inflammatory cytokines and mediators are associated with chronic pain suggesting immune-targeted approaches including the therapeutic administration of endogenous anti-inflammatory compounds could provide much needed new ways to treat, manage, and control the growing global pandemic of chronic pain.

## Introduction

The International Society for the Study of Vulvovaginal Diseases defines localized provoked vulvodynia (LPV) as vulvar pain lasting at least 3 months without readily detectable accompanying inflammation or infection (Bornstein et al., 2016). While LPV affects 8-28% of cis-gender, female-identifying individuals during their lifetime (Reed et al., 2004; Reed et al., 2012; Harlow et al., 2014), it is frequently misdiagnosed and lacks effective therapeutic approaches (Merlino et al., 2022). However, self-reported history of allergies to pollen and insect venom, and recurrent vulvovaginal yeast infections are both associated with an increased incidence of vulvodynia (Sarma et al., 1999; Witkin et al., 2002; Harlow et al., 2009; Marfatia et al., 2016; Harlow et al., 2023) suggesting that immune mechanisms may be involved in its underlying pathophysiology. Pain has been recognized as a cardinal sign of protective inflammation since at least 50 CE (in *De Medicina* by Roman encyclopedist Aulus Celsus) and is key to both healing, and withdrawal from harmful exposures. However, long-lasting inflammation can lead to dysfunctional tissue remodeling and/or central nervous sensitization producing unresolved pathological pain (Ren and Dubner, 2010). Fibroblasts derived from the vestibular tissues of vulvodynia patients showed amplified inflammatory responses to yeast antigens *in vitro* (Falsetta et al., 2015). Repeated yeast infections (Awad-Igbaria et al., 2022) and multiple exposures to irritant allergens (Landry et al., 2017) or a common chemical preservative (Arriaga-Gomez et al., 2019; Kline et al., 2020) produced persistent vulvar sensitivity to pressure along with mast cell accumulation and hyperinnervation in rodent models of vulvodynia. In this review, we discuss these studies and other lines of evidence implicating innate sensing and dysregulated immune responses in the pathology of vulvodynia and other chronic pain conditions.

## Altered fibroblast inflammatory responses to yeast are associated with vulvar pain

Fibroblast cell lines derived from LPV patients have revealed an intricate cascade of immune mechanisms underlying vulvar pain. Early studies showed higher levels of inflammatory cytokine production when these cells were stimulated with yeast antigens *in vitro*, as well as a correlation of increased interleukin (IL-6) and prostaglandin E2 (PGE2) levels with pain sensitivity (Foster et al., 2007; Foster et al., 2015).

More recent studies have attributed these differences to increased expression of pathogen recognition receptor Dectin-1 and subsequent Nuclear Factor Kappa B (NFκB) activation (Falsetta et al., 2015) as well as an increase in bradykinin receptors in LPV patients (Falsetta et al., 2016). Critical fungal recognition molecules - Toll-like Receptors (TLRs) - were more abundant in patient fibroblasts from painful tissues, and these cells produced more IL-6 upon stimulation with imiquimod agonist (Falsetta et al., 2018). These findings suggest that enhanced, site-specific, innate immune responses to pathogens by fibroblasts may be an early mediator in LPV. LPV fibroblasts also produced more IL-6 in response to bradykinin stimulation *in vitro* (Falsetta et al., 2016).

Mast cells recognize both yeasts and hyphae of *Candida albicans via* Dectin-1 and release cytokines and other granule contents during the early immune response to fungal infection (Nieto-Patlán et al., 2015). Mast cell activation and heparin release frequently leads to upregulation of tissue bradykinins (Oschatz et al., 2011). Bornstein et al. reported elevated mast cell numbers and heparanase-driven basement membrane degradation in LPV biopsies (Bornstein et al., 2004; Bornstein et al., 2008). Recently, Awad-Igbaria et al. observed local mast cell accumulation and hyperinnervation in a recurrent zymosan-challenge vulvodynia model in rats; vulvar sensitivity, local nerve growth factor (NGF) concentrations, and hyperinnervation were all reduced following treatment with mast cell stabilizer ketotifen (Awad-Igbaria et al., 2022). Therefore, mast cells activated by yeast infection could lead to endogenous tissue bradykinin production thus contributing to increased IL-6 production by fibroblasts and pain sensitivity in LPV.

## Local immune responses to allergens can also lead to prolonged vulvar pain

Skin allergies, allergies to insect venom, and seasonal allergies are all associated with an elevated risk of LPV (Harlow et al., 2009). Mast cells are key players in allergy (Galli and Tsai, 2010), and have been shown to contribute to protective pain responses in mice (Chatterjea et al., 2012) which they do in an antigen-specific manner in allergy-provoked hyperalgesia (Mack et al., 2014). We leveraged the epidemiological association of vulvodynia and allergies, and the link between mast cells and pain to establish mouse models of allergy-driven vulvodynia.

Repeated topical exposures to oxazolone (a hapten irritant that triggers contact hypersensitivity in a mast cell-dependent manner) daily for 10 days on the labial skin of previously sensitized female ND4 mice provoked tactile peri-vaginal pain responses that lasted days to weeks after overt inflammation resolved (Martinov et al., 2013; Landry et al., 2017). Accumulation of mast cells and overgrowth of CGRP^+^ neurons in these mice phenocopied the earlier results seen in LPV biopsies (Bornstein et al., 2004; Bornstein et al., 2008).

To demonstrate that these findings were not oxazolone-specific, we used a different hapten, dinitrofluorobenzene (DNFB), that we applied daily for 10 days to the vaginal canal of previously sensitized mice (Boo et al., 2019) and reproduced lasting pain sensitivity as well as mast cell accumulation in the mucosal tissues of the vaginal canal. Both oxazolone and DNFB-driven immune responses also included elevated levels of circulating Immunoglobulin E (IgE), and accumulation of CD25^+^ regulatory CD4^+^ T cells and IFN-γ producing memory CD8^+^ T cells in the affected tissues. Notably, therapeutic local mast cell depletion in both oxazolone and DNFB-driven pain by injections of mast cell degranulating compound 48/80 (c48/80) and topical application of tetrahydrocannabinol (THC) respectively reduced pain responses strengthening the case for mast cell-driven immune changes in perpetuating long-term maladaptive vulvar sensitivity.

## Immune responses to common environmental toxins may trigger vulvar pain

Reed and colleagues found a positive association between vulvodynia and the reported history of exposures to a variety of household and work-related environmental toxins (Reed et al., 2019). Isothiazolinone preservatives, common in personal care and cleaning products, are frequently linked to allergic reactions (Deza and Giménez-Arnau, 2017). >1% of people in several European countries were found to be sensitized to methylisothiazolinone (MI) which was subsequently regulated out of leave-on cosmetics in the European Union (Lundov et al., 2011; Castanedo-Tardana and Zug, 2013; Yu et al., 2016) and also named allergen of the year by the American Contact Dermatitis Society in 2013 (Castanedo-Tardana and Zug, 2013).

We found that dermally sensitized mice re-exposed to MI in the vaginal canal daily for 10 days showed local mast cell accumulation, and developed peri-vaginal sensitivity for >2 weeks after the last exposure (Arriaga-Gomez et al., 2019); both preventive and therapeutic administration of THC in the vaginal canal reduced mast cell numbers and pain sensitivity in concordance with our previous observations with contact irritants more commonly used in the laboratory. In a follow-up study, repeated daily MI exposures for 10 days on the labial skin of previously sensitized mice resulted in mast cell accumulation and remarkably persistent tactile sensitivity for up to 70 days, long past the resolution of overt inflammation indicating possible central sensitization of the nervous system. Intraperitoneal treatment with imatinib, which targets protein kinase c-Kit expressed on mast cells, prevented mast cell accumulation and abrogated tactile sensitivity (Kline et al., 2020). We derived vaginal fibroblast lines from sensitized mice exposed to 10 applications of MI or saline vehicle in the vaginal canal; fibroblasts derived from MI-challenged canals produced higher levels of IL-6 compared to those derived from saline-challenged canals when exposed to bacterial lipopolysaccharides or zymosan *in vitro*, mirroring the behavior of fibroblasts derived from vulvodynia patients (Blum et al., 2020).

The association between allergic immune responses to environmental toxins and vulvar pain in these experiments reiterates the contribution of mast cells to these pain processes and raises the possibility that exposure to other chemical preservatives might have similar outcomes. The biological plausibility we provide for the association between exposures to common chemical preservatives and chronic vulvar pain suggests the need for preventive interventions such as consumer awareness, avoidance of products containing such preservatives, and regulatory action to replace these chemicals with safer alternatives.

## Mast cell immune responses are a key orchestrator of chronic pain in vulvodynia

**Figure 1:**
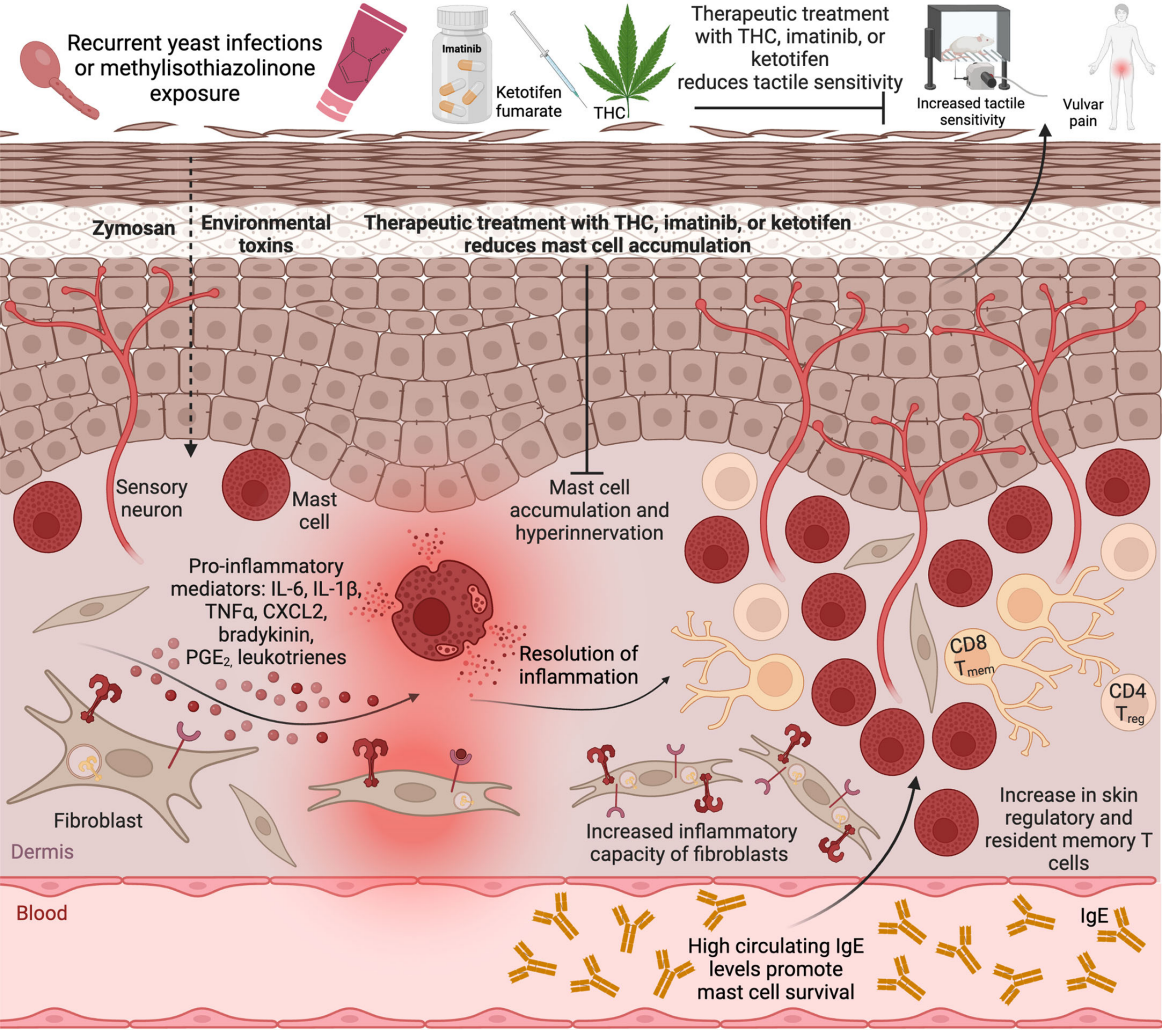
Mast cell accumulation in the tissue drives vulvar sensitivity. Repeated exposure to a common environmental toxin MI or recurrent vulvovaginal yeast infections lead to activation of local mast cells and fibroblasts and release of inflammatory cytokines accompanied by increased tactile sensitivity and pain. Upon resolution of acute inflammation, the tactile sensitivity remains, accompanied by hyperinnervation, increased mast cell numbers, and infiltration of regulatory T cells and resident memory T cells into the tissue, as well as elevated inflammatory potential of local fibroblasts. Therapeutic treatment with imatinib or THC, or preventative treatment with ketotifen, reduce mast cell accumulation and abrogate vulvar pain. Created with BioRender.com.

It is notable that the biological mechanisms linking both major risk factors - yeast infections, and a history of allergies - for vulvodynia involve innate immune responses that are likely mediated by tissue mast cells (**Figure 1**). Mast cells serve as tissue sentinels and a first line of inflammatory response in infection and allergy. Activated mast cells can stimulate fibroblasts to maintain an enhanced inflammatory profile via bradykinin signaling. IL-6 produced by activated fibroblasts can in turn promote mast cell maturation from tissue infiltrating precursors (Conti et al., 2002). Prolonged allergen exposures lead to increases in regulatory and memory T cells in affected tissues. Regulatory T cells can recruit mast cells to allergic tissue (Jones et al., 2010). IFN-γ produced by infiltrating memory T cells and circulating IgE produced by B cells can both promote mast cell survival (Yu et al., 2011; Bax et al., 2012) the latter also acting via IL-6.

Mast cells and nerves are physically co-located in many tissues, and bi-directional communication between mast cells and nerves are key to both physiological homeostatic processes and disease mechanisms (reviewed in Forsythe and Bienenstock, 2012). Mast cells interact with the nervous system through a variety of mechanisms including degranulation, *de novo* synthesis of neuro-modulatory molecules, extracellular vesicles, tunneling nanotubes, and extracellular traps (Mittal et al., 2019). Mast cell-derived NGF and Cell Adhesion Molecule 1 (CADM1) can potentially help maintain the hyperinnervation observed in clinical and pre-clinical studies of painful vulvar tissue. Peripheral nerves and mast cells may engage in a “vicious cycle” of mutual activation through release of neuropeptides, tryptase, and histamine that can cause both peripheral sensitization and a transition from acute to chronic pain (Gupta and Harvima, 2018). Action potentials generated in sensitized nerve terminals can travel to the dorsal horn resulting in activation of second order nociceptive neurons and ultimately to central sensitization and long-term pain (Gupta and Harvima, 2018).

Taken together, these findings suggest that increases in numbers of mast cells can serve both as useful biomarkers and therapeutic targets for dysregulated immune responses in vulvodynia. Mast cell-mediated inflammatory and tissue repair responses also play similar roles in other chronic pain conditions as observed in clinical samples and pre-clinical disease models discussed below.

## Mast cell increases and activation are associated with many other chronic pain conditions

Degranulation of dural mast cells by systemic c48/80 administration activated meningeal nociceptors via Tumor Necrosis Factor α (TNF-α), IL-1 β, and IL-6 via central sensitization mechanisms in a rat model of migraine pain (Levy et al., 2007; Zhang et al., 2011; Zhang et al., 2012). Mast cell-deficient mice exhibited less painful sensitivity in a model of sickle-cell anemia (Vincent et al., 2013). Tissue from patients with chronic painful pancreatitis showed increased interstitial mast cells and the finding was corroborated by lower tactile sensitivity in mast-cell deficient mice in 2,4,6 trinitrobenzene sulfonic acid (TNBS)-induced chronic pancreatitis (Hoogerwerf et al., 2005). Mast cell numbers and degranulation were increased in endometriosis lesions relative to controls and these findings were replicated in a surgically induced rat model of endometriosis (Anaf et al., 2006).

Prostatic secretions from patients with chronic pelvic pain syndrome had increased numbers of degranulated mast cells and higher NGF levels; mast cell-deficient mice had a higher threshold for lower abdominal pain driven by immune messengers CCL2 and CCL3 in a model of experimental autoimmune prostatitis (Done et al., 2012; Quick et al., 2012). CCL2 mediated tactile sensitivity in the suprapubic and hind paw regions of female mice through mast cell recruitment to the bladder in a model of autoimmune cystitis (Bicer et al., 2015). CCL2 induced mast cell degranulation and histamine release (Lv et al., 2012) in mouse interstitial cystitis where histamine mediated tactile sensitivity, and TNF-α mediated bladder pathophysiology (Rudick et al., 2008; Rudick et al., 2009).

Mast cells are important in tissue repair after injury or acute inflammatory events (Galli and Tsai, 2008), and this tissue remodeling can potentially alter pain sensitivity. In patellar tendinopathy - an overuse-triggered pain condition - mast cells accumulated in the joint, signaled tendon-specific fibroblasts to increase production of matrix metalloproteinases, and contributed to tendon degradation via matrix remodeling of tendon tissue (Behzad et al., 2013). IL-1β and Substance P-mediated mast cell accumulation and degranulation were found to be necessary for post-fracture mechanical allodynia in Complex Regional Pain Syndrome in rats (Zhang et al., 2022).

## Innate immune cells and mediators yield new targets and strategies for chronic pain management

As a cardinal sign of inflammation, pain is initially protective; it signals the detection of harmful stimuli and induces withdrawal and lack of use that allows healing (Woolf, 2010). Innate immune myeloid cells, including macrophages, neutrophils, and mast cells are activated by pathogens (Stegelmeier et al., 2019) and tissue damage (del Fresno and Sancho, 2021). Activated immune cells and fibroblasts release inflammatory cytokines and chemokines that contribute to acute hypernociception (Verri et al., 2006).

Innate cells including neutrophils, mast cells, and macrophages can also regulate the transition of acute to chronic pain, and contribute to its maintenance via complex neuroimmune signaling (Yang et al., 2022) that is often highly context-specific. For example, an early acute neutrophil response assessed by transcriptomic analysis was associated with lower chronic back pain in a sample of 98 adults; early depletion of neutrophils delayed pain resolution in mice and exogenous administration of neutrophils or neutrophil products prevented long lasting pain (Parisien et al., 2022). On the other hand, neutrophils were shown to be the primary drivers of chronic widespread pain in a recent mouse model of fibromyalgia (Caxaria et al., 2023).

One mouse model of vaginal candidiasis-driven vulvar pain reported no increase in any immune cells in the affected tissue (Farmer et al., 2011), in line with some clinical vulvodynia studies that also reported no changes in mast cells (Papoutsis et al., 2016). While these studies may have missed a transient mast cell spike (as several other clinical biopsies (reviewed in Chalmers et al., 2016) did reveal increased mast cells numbers), mast cells are likely not the only instigators of vulvar pain. Treatment with macrophage-targeting clodronate reduced vaginal M1 and M2 macrophages and visceromotor responses to vaginal distension caused by microinjection of Complete Freund’s Adjuvant (CFA) (Castro et al., 2022). A different model of vestibular CFA injection-driven vestibular pain led to increases in renin-angiotensin system proteins with both macrophages and T cells contributing to increased renin and angiotensinogen (Chakrabarty et al., 2018).

Despite these exceptions, as shown in multiple pre-clinical studies of vulvodynia discussed above (Arriaga-Gomez et al., 2019; Kline et al., 2020; Awad-Igbaria et al., 2022), therapies that reduced mast cell accumulation and activation also abrogated vulvar pain. Falsetta and colleagues found that injecting exogenous pro-resolving mediators reduced prostaglandin E2 production in fibroblasts from vulvodynia biopsies (Falsetta et al., 2021). Specialized pro-resolving lipid mediators (SPM) such as maresins are converted from omega-6-polyunsaturated fatty acids by immune cells, fibroblasts, epithelial and endothelial cells, and downregulate inflammation in a plethora of diseases including asthma, arthritis, ischemia-reperfusion injury, and acute inflammatory pain (Yang et al., 2021).

Thus, targeting specific, relevant, innate immune cellular and molecular components in ways that minimize immune suppression and maximize control of pathological inflammation can provide novel therapeutic approaches for vulvodynia and other chronic pain conditions.

## Conclusion

Vulvodynia affects quality of life in substantive and deleterious ways including sexual dysfunction, infertility, depression, and compromised mobility. A survey of 280 patients at University of California, San Francisco found that of all vulvar conditions included in the survey, a diagnosis of vulvodynia was the most strongly related to a poor quality of life, and effects on physical and social functions were worse than for other vulvar pathologies (Ponte et al., 2009). Despite its high incidence among the cis-gender women represented in the large and medium-scale epidemiological studies (Reed et al., 2012; Harlow et al., 2014), no effective medical therapy exists for vulvodynia. Researchers and clinicians should therefore pay close attention to our collective, evolving understanding of how immune mechanisms mediated by mast cells, fibroblasts and other cells contribute to chronic pain, and use these discoveries to develop new ways to treat and manage this challenging condition. Immune targeted therapies have the potential to transform the treatment and management of vulvodynia, and also provide much needed additions to the toolkits of therapeutic approaches for many other chronic pain conditions.

## Author contributions

GO, KT, and XMG reviewed the literature and drafted summaries. ET and DC wrote the manuscript. ET prepared the figure and edited the manuscript. DC conceived, designed, and supervised the manuscript writing. All authors contributed to the article and approved the submitted version.
